# Correspondence of Somatic Cell Counts in Bulk-Tank Milk to Prevalence of Subclinical Mastitis in Sheep Flocks

**DOI:** 10.3390/ani13223541

**Published:** 2023-11-16

**Authors:** George C. Fthenakis

**Affiliations:** Veterinary Faculty, University of Thessaly, 43100 Karditsa, Greece; gcf@vet.uth.gr

**Keywords:** goat, mastitis, milk, predictor, prevalence, sheep, somatic cell counts, *Staphylococcus*, subclinical mastitis, vaccination

## Abstract

**Simple Summary:**

This work presents an analysis of data for inferring the prevalence of subclinical mastitis in sheep flocks through the somatic cell counts in the bulk-tank milk of farms. Cell counts in bulk-tank milk between 0.100 × 10^6^ and 0.400 × 10^6^ cells mL^−1^ were considered to correspond to prevalence of the infection between 8.7% and 12.1%, cell counts between 0.400 × 10^6^ and 0.650 × 10^6^ cells mL^−1^ corresponded to prevalence between 12.4% and 19.4%, cell counts between 0.650 × 10^6^ and 900 × 10^6^ cells mL^−1^ corresponded to prevalence of the infection between 22.5% and 30.8% and cell counts between 0.900 × 10^6^ and 1.450 × 10^6^ cells mL^−1^ corresponded to prevalence between 27.3% and 45.3%. The information that may be thus obtained can be useful in guiding the implementation of various health management procedures for mastitis control in sheep flocks.

**Abstract:**

The objective of the present study was to propose thresholds of somatic cell counts in bulk-tank milk indicative of the prevalence of subclinical mastitis in a flock. A retrospective analysis was performed on data from a longitudinal survey of subclinical mastitis in Greece, in which the prevalence of subclinical mastitis in 12 flocks sampled four times throughout a milking period was evaluated by collecting milk samples from individual ewes for bacteriological and cytological testing; further, cell counts in the bulk-tanks of the farms were also measured during the visits. Four cohorts were created: A, with cell counts in the bulk-tank milk between 0.100 × 10^6^ and 0.400 × 10^6^ cells mL^−1^, B, with cell counts between 0.400 × 10^6^ and 650 × 10^6^ cells mL^−1^, C, with cell counts between 0.650 × 10^6^ and 900 × 10^6^ cells mL^−1^, and D, with SCC between 0.900 × 10^6^ and 1.450 × 10^6^ cells mL^−1^. There was a significant positive correlation between prevalence of the infection in the flocks and somatic cell counts in bulk-tank milk on the same sampling occasion (*p* < 0.0001). There was also evidence of significant differences between the four cohorts in the mean prevalence rate of the infection (*p* < 0.0001). Ninety-five percent confidence intervals of the prevalence of subclinical mastitis according to the somatic cell counts in the bulk-tank milk were calculated as follows: for cohort A, 8.7% to 12.1%, for B, 12.4% to 19.4%, for C, 22.5% to 30.8% and for D, 27.3% to 45.3%. The information that may be thus obtained can be useful in guiding the implementation of various health management procedures for mastitis control in sheep flocks, with no need to perform milk sample collection from ewes and subsequent laboratory examinations.

## 1. Introduction

In dairy cattle, the evaluation of somatic cell counts in bulk-tank milk, in order to infer information regarding the prevalence of subclinical mastitis in herds, has been applied since the 1980s [1,2].

However, in contrast, there is little relevant evidence in sheep. The first researchers to study this issue were Berthelot et al. [3], who proposed that 0.650 × 10^6^ cells mL^−1^ in the bulk-tank milk of sheep flocks corresponded to 15% prevalence of subclinical mastitis in the respective flocks. Thereafter, Leitner et al. [4] suggested that 0.800 × 10^6^ cells mL^−1^ in the bulk-tank milk corresponded to 25% prevalence of subclinical mastitis in the respective flock, with higher cell counts (1.500 × 10^6^ cells mL^−1^) indicating a higher prevalence (up to 50%).

The objective of the present study was to propose thresholds of somatic cell counts in bulk-tank milk indicative of and corresponding to the prevalence of subclinical mastitis in the flock.

## 2. Materials and Methods

The work is a retrospective analysis study of data from a longitudinal survey of subclinical mastitis in Greece [5]. A brief description of that study follows herein. Twelve dairy sheep flocks were included in the study and were visited four times during a 6- to 6.5-month-long milking period. During each visit, mammary secretion samples were collected from both mammary glands of the same 20, at least secundiparae, ewes in each flock; moreover, samples of milk were also collected at the same time from the bulk tank of the farm.

Hence, the study involved 240 ewes, which were examined four times throughout their milking period; thus, 948 samplings of ewes with clinically normal udders were performed during the study and therefore 1896 mammary secretion samples were collected from individual ewes. Moreover, the study involved 12 flocks, from which bulk-tank milk samples were obtained, with quadruplicate samples collected on each sampling occasion; thus, 48 samplings of farm bulk tanks were performed during the study and therefore 192 bulk-tank milk samples were collected from the sheep flocks.

Established microbiological examinations were performed on mammary secretion samples in order to isolate mastitis-causing pathogens. Further, cytological examinations (somatic cell counting and California Mastitis Test) were conducted to measure the cellular content in all the samples (mammary secretion samples and bulk-tank milk).

### 2.1. Data Management and Analysis

#### 2.1.1. Data Management

The definition of subclinical mastitis was based on a combination of bacteriological and cytological findings, always in the absence of clinically evident findings as defined previously and as described in detail in Table S1. Staphylococcal subclinical mastitis was defined as when *Staphylococcus* spp. was identified as the causal pathogen of subclinical mastitis.

Sampling occasions were divided into four cohorts, using as a criterion and in accordance to the somatic cell counts in the bulk-tank milk. These cohorts were as follows: cohort A included sampling occasions on which somatic cell counts in the bulk-tank milk were from 0.101 × 10^6^ to 0.400 × 10^6^ cells mL^−1^, cohort B included sampling occasions on which somatic cell counts in the bulk-tank milk were from 0.401 × 10^6^ to 0.650 × 10^6^ cells mL^−1^, cohort C included sampling occasions on which somatic cell counts in the bulk-tank milk were from 0.651 × 10^6^ to 0.900 × 10^6^ cells mL^−1^ and cohort D included sampling occasions on which somatic cell counts in the bulk-tank milk were from 0.901 × 10^6^ to 1.500 × 10^6^ cells mL^−1^.

The prevalence rate of the infection (subclinical mastitis or staphylococcal subclinical mastitis) was calculated on each sampling occasion as the proportion of the ewes found with the infection upon evaluation among the ewes that were sampled on that occasion.

#### 2.1.2. Statistical Analysis

All data were entered into Microsoft Excel and analyzed using IBM SPSS Statistics (ver. 21) (IBM; Armonk, NY, USA). Initially, a Spearman rank correlation analysis was performed between the prevalence of subclinical mastitis or staphylococcal subclinical mastitis and the somatic cell counts in the bulk-tank milk on each sampling occasion. A *z*-test on Fisher *z*-transformed correlation coefficients [6] was employed to assess any significant difference between the two correlation coefficients.

The prevalence rates of each infection (subclinical mastitis or staphylococcal subclinical mastitis) of the four cohorts were compared between them by using analysis of variance; this was followed by Tukey’s significant difference calculation. Thereafter, 95% confidence intervals were calculated for the prevalence of each infection (subclinical mastitis or staphylococcal subclinical mastitis) for samplings occasions within each cohort and according to the somatic cell counts in the bulk-tank milk.

In all analyses, statistical significance was defined at *p* < 0.05.

## 3. Results

**Figure 1:**
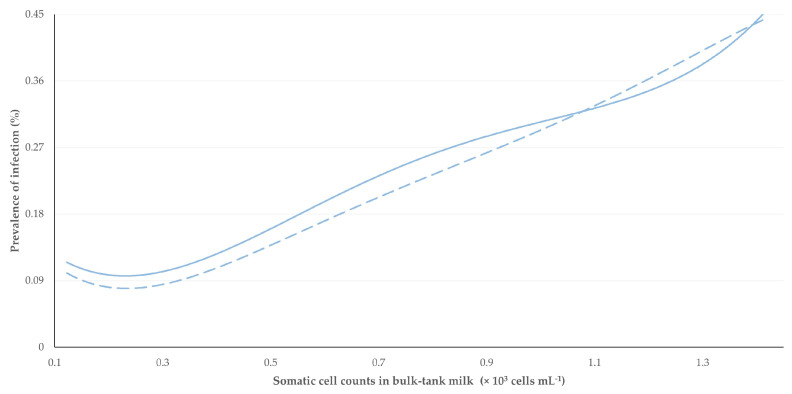
Trendlines of correlation between prevalence of subclinical mastitis (solid line)/staphylococcal subclinical mastitis (dashed line) and somatic cell counts in bulk-tank milk on the same sampling occasion.

There was a significant positive correlation between the prevalence of subclinical mastitis or staphylococcal subclinical mastitis and somatic cell counts in bulk-tank milk: *r*_sp_ = 0.711 and 0.741, respectively (*p* < 0.0001) (Figure 1). The difference between the two correlation coefficients was not significant: |*z|* = 0.300 (*p* = 0.76).

**Figure 2:**
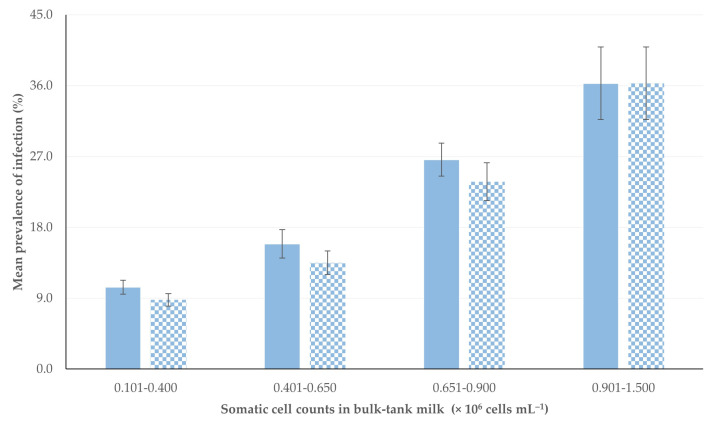
Mean prevalence rate of subclinical mastitis (solid fill)/staphylococcal subclinical mastitis (pattern fill), according to the somatic cell counts in the bulk-tank milk on the respective sampling occasion.

There were also significant differences in the mean prevalence rates of subclinical mastitis and staphylococcal subclinical mastitis between the four cohorts of sampling occasions, i.e., according to the somatic cell counts in the bulk-tank milk (*p* < 0.0001 between the four cohorts for both infections) (Table 1, Figure 2).

The calculated 95% confidence intervals for the prevalence of subclinical mastitis and staphylococcal subclinical mastitis according to the somatic cell counts in the bulk-tank milk showed an interlap only between the third and the fourth cohort, i.e., among sampling occasions with somatic cell counts from 0.651 × 10^6^ to 0.900 × 10^6^ cells mL^−1^ and from 0.901 × 10^6^ to 1.500 × 10^6^ cells mL^−1^ (Table 2).

**Figure 3:**
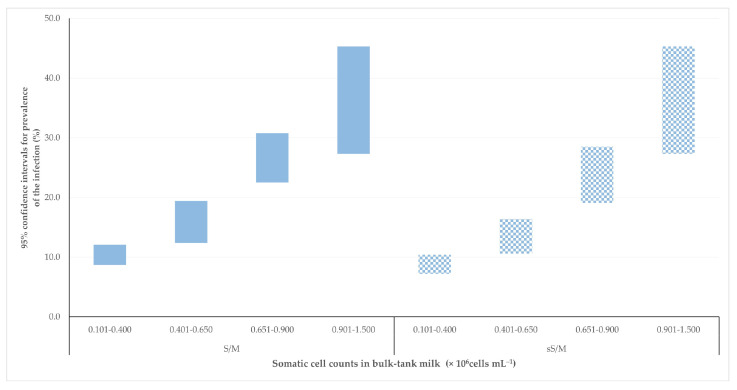
Calculated 95% confidence intervals for prevalence of subclinical mastitis (S/M, solid fill)/staphylococcal subclinical mastitis (sS/M, pattern fill), according to the somatic cell counts in the bulk-tank milk.

There was no significant difference in the width of the calculated confidence intervals between subclinical mastitis and staphylococcal subclinical mastitis: the median values (interquartile range) were 7.7 (4.6) and 7.6 (6.4), respectively (*p* = 0.99). Moreover, whilst the interlap noted in third and fourth intervals was more narrow for staphylococcal subclinical mastitis, 1.2 percentage points (3.3% of cumulative confidence intervals) versus 3.5 percentage points (9.5% of cumulative confidence intervals) for subclinical mastitis, the difference was not significant (*p* = 0.28) (Figure 3).

## 4. Discussion

Mastitis, a disorder adversely affecting the welfare of sheep [7], is the most important and significant factor leading to increased somatic cell counts in milk. The cumulative evidence from the international literature [8] confirms that somatic cell counts in bulk tanks depend significantly on the presence of mastitis in a flock, whilst other factors have been found to be of lesser significance. Specifically in Greece, in a recent field study carried out in the country, dairy sheep flocks where farmers reported an annual incidence risk of clinical mastitis >0.5% were found to have significantly higher somatic cell counts in the bulk-tank milk [9].

Whilst clinical mastitis is easy to identify and diagnose, subclinical mastitis requires laboratory examinations, specifically a combination of microbiological and cytological tests for definite diagnosis [9]. This is the reason that the somatic cell counts of the bulk-tank milk are used as a proxy to reveal the presence and the extent of subclinical mastitis on dairy cattle farms.

The present communication contributes to establishing this approach on dairy sheep farms as well. The present retrospective analysis study provides intervals for the correspondence of somatic cell counts in bulk-tank milk to the prevalence of subclinical mammary infection in the flock on the same sampling occasion. The findings of the analysis are in line with both previous relevant studies carried out by other researchers. The findings reveal an accord for low somatic cell counts in the bulk-tank milk, agreement with the previous finding of 0.650 × 10^6^ cells mL^−1^ in the bulk-tank milk of sheep flocks corresponding to 15% prevalence of subclinical mastitis [3], and for high somatic cell counts in the bulk-tank milk, agreement with the previous finding of 1.500 × 10^6^ cells mL^−1^ in the bulk-tank milk of sheep flocks corresponding to 50% prevalence of subclinical mastitis [4].

The current findings further the previous suggestions and provide more detailed classification of somatic cell counts in the bulk-tank milk for correspondence with the prevalence of subclinical mammary infection. This supports better inference of the frequency of the infection in a flock. It is noted that legal thresholds for the upper level of somatic cell counts in sheep milk produced for human consumption have not been defined [10,11], which points to the necessity to produce scientific information for use in sheep farms.

There is merit in the information obtained in the present study, as this can be useful for guiding the implementation of various health management procedures for the control of mastitis in sheep flocks, without the need to perform repeated milk sample collection from ewes and subsequent laboratory examination. For example, an indication of increased prevalence of staphylococcal subclinical mastitis in the flock could support a decision to vaccinate ewes against the infection [9].

## 5. Conclusions

This paper provides a model for inferring the prevalence of subclinical mastitis in sheep flocks through somatic cell counts in bulk-tank milk, based on the results of a field investigation. The findings are in agreement with the results of previous relevant studies, which were of more limited scope and extent [3,4]. The current study complements and extends the previous findings.

In the present study, cell counts in the bulk-tank milk between 0.100 × 10^6^ and 0.400 × 10^6^ cells mL^−1^ were considered to correspond to prevalence of the infection between 8.7% and 12.1% in the flock, cell counts between 0.400 × 10^6^ and 0.650 × 10^6^ cells mL^−1^ corresponded to prevalence between 12.4% and 19.4% in the flock, cell counts between 0.650 × 10^6^ and 900 × 10^6^ cells mL^−1^ corresponded to prevalence of the infection between 22.5% and 30.8% in the flock and cell counts between 0.900 × 10^6^ to 1.450 × 10^6^ cells mL^−1^ corresponded to prevalence between 27.3% and 45.3% in the flock.

## Figures and Tables

**Table 1:** Mean (±standard error of the mean) prevalence rate of subclinical mastitis/staphylococcal subclinical mastitis according to the cell counts in the bulk-tank milk on the respective sampling occasion.

Somatic Cell Counts on Respective Sampling Occasions	Prevalence of Infection	
	Subclinical Mastitis	Staphylococcal Subclinical Mastitis
0.101 × 10^6^ to 0.400 × 10^6^ cells mL^−1^	10.4% ± 0.9% ^a,b^	8.8% ± 0.8% ^a,b,c^
0.401 × 10^6^ to 0.650 × 10^6^ cells mL^−1^	15.9% ± 1.8% ^c,d^	13.5% ± 1.5% ^a,d,e^
0.651 × 10^6^ to 0.900 × 10^6^ cells mL^−1^	26.6% ± 2.1% ^a,c^	23.8% ± 2.4% ^b,d^
0.901 × 10^6^ to 1.500 × 10^6^ cells mL^−1^	36.3% ± 4.6% ^b,d^	36.3% ± 4.6% ^c,e^

^a–e^: within the same column, *p* < 0.05 for differences between respective cohorts.

**Table 2:** Calculated 95% confidence intervals for prevalence of subclinical mastitis/staphylococcal subclinical mastitis according to the somatic cell counts in the bulk-tank milk.

Somatic Cell Counts on Respective Sampling Occasions	Prevalence of Infection	
	Subclinical Mastitis	Staphylococcal Subclinical Mastitis
0.101 × 10^6^–0.400 × 10^6^ cells mL^−1^	8.7–12.1%	7.2–10.4%
0.401 × 10^6^–0.650 × 10^6^ cells mL^−1^	12.4–19.4%	10.6–16.4%
0.651 × 10^6^–0.900 × 10^6^ cells mL^−1^	22.5–30.8%	19.1–28.5%
0.901 × 10^6^–1.500 × 10^6^ cells mL^−1^	27.3–45.3%	27.3–45.3%

## Data Availability

Retrospective analysis data presented by Michael et al. [5].

## Supplementary Materials

The following are available online at https://www.mdpi.com/article/10.3390/ani13223541/s1, Table S1: Detailed description of the criteria for definition of subclinical mastitis in sheep flocks. Reference [12] are cited in the supplementary materials.
