# Supplementary material for: Correspondence of Somatic Cell Counts in Bulk-Tank Milk to Prevalence of Subclinical Mastitis in Sheep Flocks
Source: Animals (Basel). 2023 Nov 16;13(22):3541. doi: 10.3390/ani13223541 (PMC10668708; doi:10.3390/ani13223541)
Supplement: Supplementary file 1 [file animals-13-03541-s001.zip › animals-2703459-SI.pdf]

# Correspondence of Somatic Cell Counts in Bulk-Tank Milk with Prevalence of Subclinical Mastitis in Sheep Flocks

George C. Fthenakis

Table S1. Detailed description of the criteria for definition of subclinical mastitis in sheep flocks.

Subclinical mastitis was defined in ewes, in which:
(1) a bacteriologically positive mammary secretion sample: [a] > 10 colonies of the same organism and [b] no more than two different types of colonies,
(2) with concurrently increased cell content: [a] CMT score ≥ '1' and [b] neutrophil and lymphocyte proportion cumulatively ≥ 65% of all leucocytes, was detected,
(3) with no presence of abnormal gross findings in the mammary gland (including changes in secretion) [Vasileiou et al. 2018].

Reference

Vasileiou, N.G.C.; Cripps, P.J.; Ioannidi, K.S.; Chatzopoulos, D.C.; Gougoulis, D.A.; Sarrou, S.; Orfanou, D.C.; Politis, A.P.; Calvo Gonzalez-Valerio, T.; Argyros, S. et al. Extensive countrywide field investigation of subclinical mastitis in sheep in Greece. *J. Dairy Sci.* **2018**, *101*, 7297–7310
